# An unusual case of spinal cord compression secondary to iliopsoas cavernous hemangioma: A case report and literature review

**DOI:** 10.1097/MD.0000000000043061

**Published:** 2025-06-27

**Authors:** Tan-Si Chu, Tan-Huy Chu, Tri-Dung Huynh, Hoang-Vu Mai, Van-Dinh Phan, Bao-Ngoc Dang, Quoc-Dat Tran, Xuan-Sang Le, Duy-Quang Phan, Anh-Dung Nguyen, Dinh-Hoan Duong

**Affiliations:** a Department of Neurosurgery, Tam Anh Hospital, Ho Chi Minh City, Vietnam; b Tam Anh Research Institute, Ho Chi Minh City, Vietnam; c Department of Vascular Surgery, Tam Anh Hospital, Ho Chi Minh City, Vietnam; d Department of Endovascular Radiology, Tam Anh Hospital, Ho Chi Minh City, Vietnam.

**Keywords:** angiography, cavernous hemangioma, embolization, neurosurgery, vascular malformation

## Abstract

**Rationale:**

We report on a 16-year-old woman who had a history of a vascular malformation involving the left iliopsoas muscle and underwent multiple sclerotherapy sessions over the preceding 5 years without clinical improvement.

**Patient concerns:**

Ten days before admission, the patient experienced the acute onset of paraparesis. Computed tomography and magnetic resonance imaging revealed a large vascular malformation, measured 90 × 100 × 100 mm, situated at the T12 to L4 vertebral levels within the left iliopsoas muscle, exerted significant compression on the conus medullaris and cauda equina.

**Diagnoses:**

Preoperative diagnosis of a vascular malformation in the left iliopsoas muscle compressed the spinal cord.

**Interventions:**

She underwent angiography and embolization of hemangioma, followed by 2 surgeries, the initial resection of intraspinal hemangioma with concurrent spinal cord decompression, and the subsequent resection of the iliopsoas hemangioma. This staged strategy was deemed a safe and efficacious approach. Intraoperative findings and subsequent histopathological analysis concluded the diagnosis of iliopsoas cavernous hemangioma.

**Outcomes:**

After 8 months of follow-up, the patient recovered completely.

**Lessons:**

To the best of our knowledge, this is the first report of cavernous hemangioma in the iliopsoas muscle compressing the spinal cord. This study aims to highlight the diagnostic complexities, the chosen therapeutic strategy, and the role of pathological evaluation in such rare entities.

## 1. Introduction

Vascular abnormalities are classified into vascular malformations and vascular tumors based on histology, biological behavior, and clinical presentation. Vascular malformations usually present at birth, grow slowly, and are infiltrative and destructive, whereas vascular tumors rarely present at birth, grow rapidly, and can be destructive.^[1,2]^ Given that the majority of vascular malformations and approximately 40% of vascular tumors necessitate intervention, and considering their inherent complexity, a multidisciplinary approach is usually necessary. Both vascular malformations and vascular tumors usually occur in the skin or subcutaneous tissues, with very few reported cases in muscular structures.^[1–7]^ Here, we reported an extremely rare case of a patient with a vascular malformation (cavernous hemangioma) in the iliopsoas muscle compressing the spinal cord, which was successfully treated with angiography, embolization, and followed by 2 surgeries. In addition, we also provide the literature review and recommendations for management and treatment.

## 2. Case presentation

We report on a 16-year-old woman who experienced a palpable, sizable mass in the left lumbar region, exhibited tenderness to palpation, and restricted range of motion. Ten days before admission, the patient experienced the acute onset of paraparesis, and the symptoms kept getting worse. She was admitted to Tam Anh Hospital. She had a medical history of vascular malformation involving the left iliopsoas muscle, and underwent multiple sclerotherapy treatments at various healthcare facilities within Vietnam over the past 5 years, with no response.

**Figure 1.**
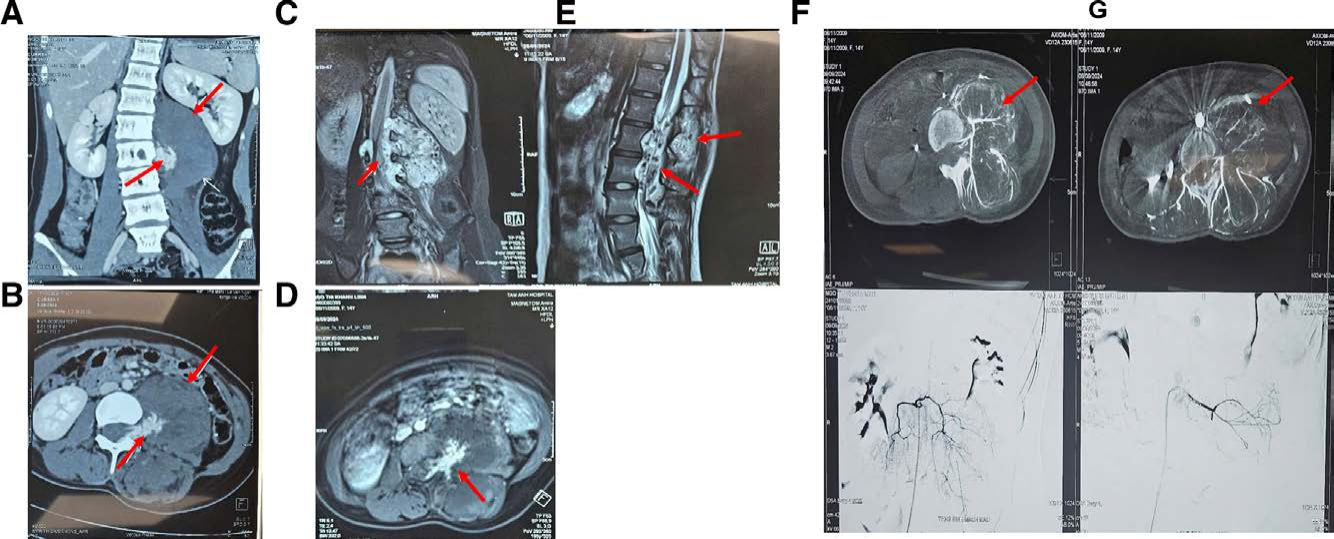
CT revealed a large vascular malformation in the iliopsoas muscle, measured 90 × 100 × 100 mm, in (A) coronal and (B) axial view. MRI revealed a large vascular malformation at the level of the T12 to L4 vertebrae in the iliopsoas muscle, compressed the conus medullaris and cauda equina, in (C) coronal, (D) axial, and (E) sagittal views. (F) Before and (G) after angiography and embolization of the vascular malformation, postintervention imaging revealed a hypervascular mass encompassed multiple paraspinal muscles and the left iliopsoas muscle, primarily fed by the left lumbar arteries at the L1 and L2 levels, underwent approximately 90% occlusion. The red arrow points at the hemangioma position. CT = computed tomography, MRI = magnetic resonance imaging.

**Figure 2.**
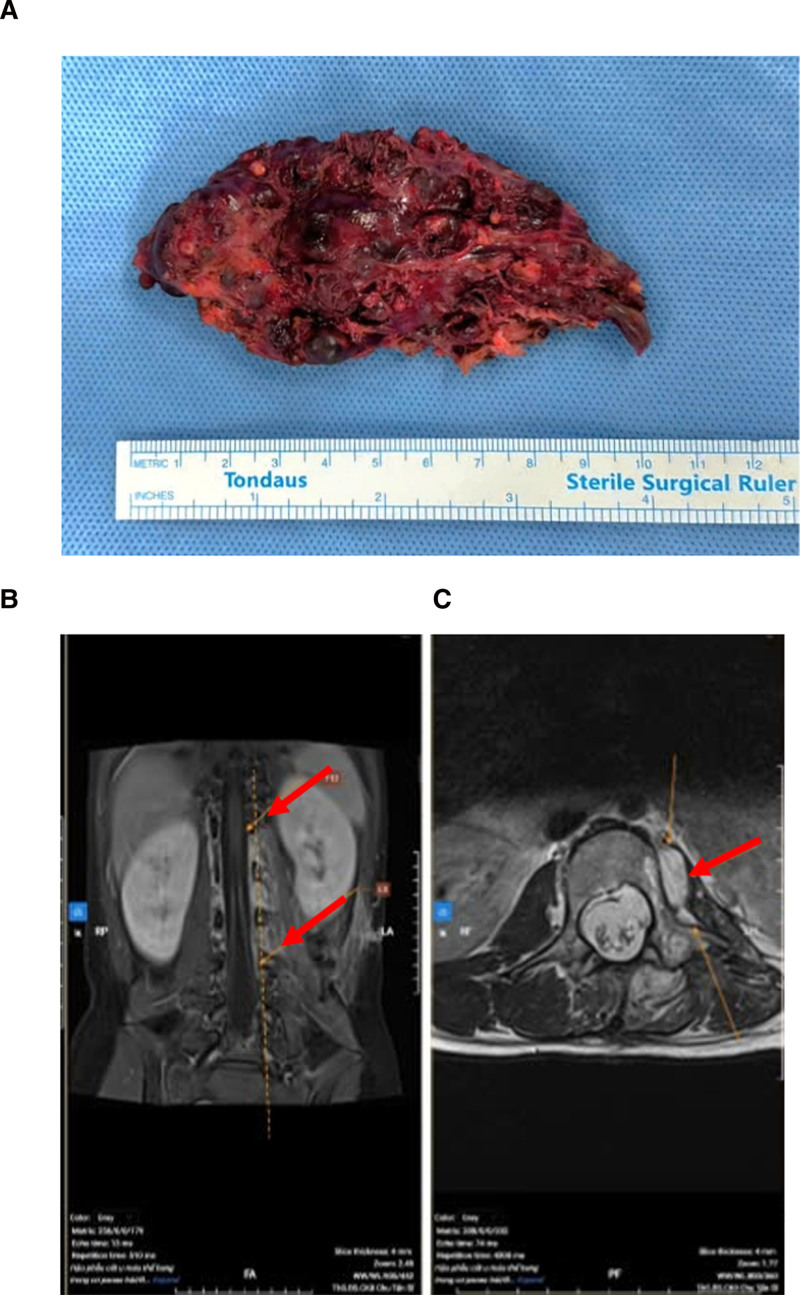
(A) The cavernous hemangioma was removed completely, measuring 100 × 80 mm. The resected specimen displayed an indurated and firm external surface with multiple superficial thrombotic projections. Internal examination revealed dilated venous channels largely filled with organized thrombi. After 4 months of follow-up, postoperative MRI revealed a significant reduction in the size of the cavernous hemangioma, with residual scattered lesions observed within multiple paraspinal muscles and the left iliopsoas muscle, spanned the T12 to L3 vertebral levels, in (B) coronal and (C) axial views. The red arrow points at the hemangioma position. MRI = magnetic resonance imaging.

On the physical examination, the blood pressure, pulse, temperature, and respiration rate were 120/70 mm Hg, 100/min, 37°C, and 20/min, respectively, the patient’s condition overall was poor. Neurological examination revealed a Glasgow Coma Scale score of 15 points, drowsy mental status, and paraparesis with hypesthesia in the lower extremities. In detail, the left and right foot muscle strength grading was both grade 1. Computed tomography (CT) and magnetic resonance imaging (MRI) revealed a large vascular malformation at the level of the T12 to L4 vertebrae in the left iliopsoas muscle, measured 90 × 100 × 100 mm, compressed the conus medullaris and cauda equina (Fig. 1A–E). The patient underwent angiography and embolization of the vascular malformation. Postintervention imaging revealed a hypervascular mass encompassed multiple paraspinal muscles and the left iliopsoas muscle, primarily fed by the left lumbar arteries at the L1 and L2 levels, underwent approximately 90% occlusion (Fig. 1F, G). One day later, the patient underwent the first surgery to remove intraspinal hemangioma with concurrent spinal cord decompression. With an approach from the lower back, we dissected the fascia and exposed the posterior spinous processes and bilateral laminae from L1 to L4, and exposed the tumor. The tumor was well-defined, compressed the back of L1 to L4 vertebral bodies, demonstrated significant vascular proliferation with intratumoral hemorrhage, and was an extra-axial tumor. Next, we dissected the tumor and released the tension of the tumor from vertebral bodies. Three days after the first operation, the patient’s condition improved with both the left and right foot muscle strength grading both grade 3, with no urinary or bowel dysfunction, and had been transitioned to a rehabilitation therapy program. Seven days after the first operation, the patient underwent the second surgery to remove the hemangioma in the iliopsoas muscle. With an approach from the left side of the back, we exposed the iliopsoas muscle and the tumor. The tumor was well-defined, measuring 100 × 80 mm, and compressed the surrounding structures, but did not invade the left kidney (Fig. 2A). The tumor was removed completely, and the pathology result concluded the diagnosis was iliopsoas cavernous hemangioma. After 4 months of follow-up, postoperative MRI revealed a significant reduction in the size of the cavernous hemangioma (Fig. 2B, C). After 8 months of follow-up, the patient recovered completely.

## 3. Discussion

A critical distinction must be made between vascular malformations and vascular tumors due to their disparate biological characteristics; the subsequent discussion will focus specifically on vascular malformations. Vascular malformation occurs due to errors during vascular genesis. The genesis of the vascular system commences around the 17th day of gestation with the development of a shared capillary plexus. Subsequently, this plexus undergoes differentiation, giving rise to capillaries, arteries, veins, and lymphatic vessels. Aberrations during this critical phase, wherein the nascent vasculature is refined and adapted, are postulated to underlie the etiology of vascular malformations. These developmental aberrations result in the formation of thin-walled vessels exhibiting a dilated lumen lined by an attenuated layer of smooth muscle cells and pericytes.^[3,8]^ In vivo studies have identified associations between various genetic abnormalities and the development of vascular malformations, implicating several genes and their cognate receptors within key signaling pathways, including TIE2, PIK3CA, Akt, RAS, GNAQ, and VEGFR2.^[9]^ The 2018 updated International Society for the Study of Vascular Anomalies categorizes vascular malformations into 4 principal groups: (1) simple vascular malformations, characterized by the involvement of a single vessel lineage, encompassing arteries (fast-flow malformations), capillaries, veins, or lymphatic vessels (slow-flow malformations); (2) combined vascular malformations, which involve 2 or more distinct vessel types within the lesion; (3) malformations affecting major named vessels; and (4) vascular malformations associated with other anomalies.^[9]^

To the best of our knowledge, this is the first report of cavernous hemangioma in the iliopsoas muscle compressing the spinal cord. Venous malformations, formerly termed cavernous hemangiomas, represent the most frequently encountered category of vascular malformations. While cavernous hemangiomas typically manifest in the skin or subcutaneous tissues, their occurrence within muscular structures is uncommon, and retroperitoneal presentation specifically within the iliopsoas muscle is exceptionally rare.^[5–7]^ Retroperitoneal cavernous hemangiomas usually remain asymptomatic in the early stage; however, the compression to nearby anatomic structure can cause nonspecific symptoms such as abdominal discomfort, hydronephrosis, dilation of the upper ureter, or even paraparesis, and hypesthesia.^[5]^ The imaging characteristics of cavernous hemangiomas are varied, while CT and MRI are commonly utilized modalities, they often provide a limited basis for definitive preoperative diagnosis. In this case, the mass caused spinal cord compression, and emergent intervention was indicated. The multidisciplinary approach is critical, especially in these rare cases,^[10,11]^ which involves angiography and embolization to optimize imaging and reduce intraoperative blood loss, followed by 2 surgeries, the initial resection of intraspinal hemangioma with concurrent spinal cord decompression, and the subsequent resection of the iliopsoas hemangioma, and also provides pathological confirmation. Pathological examination remains the gold standard for definitive diagnosis, particularly in such exceptionally rare cases.^[7,10]^ Pathologically, cavernous hemangiomas exhibit a characteristic sponge-like architecture, composed of dilated vascular lumen lined by a single layer of endothelial cells.^[5]^ Definitive diagnosis of cavernous hemangiomas is difficult and requires a thorough clinical examination, radiological features, and pathology studies to exclude the possibility of malignancy, which is important to minimize surgical and postoperative compilations.

In conclusion, cavernous hemangioma of the iliopsoas muscle poses a significant preoperative diagnostic challenge owing to its rarity and the nonspecific nature of its clinical and radiological presentation. Furthermore, when such a mass causes spinal cord compression, emergent intervention is indicated. The approach involves angiography and embolization to optimize imaging and reduce intraoperative blood loss; followed by 2 surgeries, the initial resection of intraspinal hemangioma with concurrent spinal cord decompression, and subsequent resection of the iliopsoas hemangioma, which represents a safe and efficacious strategy. This approach facilitates enhanced preoperative imaging, minimizes surgical blood loss, mitigates complications associated with spinal cord compression, provides pathological confirmation for diagnosis, and excludes malignancy. In this study, we aim to shed light on the difficulties of diagnosing these rare entities, the crucial role of therapeutic strategies employed, and the pathological assessment.

## Acknowledgments

The authors would like to especially thank Doctor Le-Tri Phuong, Tam Anh Research Institute, Ho Chi Minh City, and the staff of Tam Anh Research Institute, Ho Chi Minh City, Vietnam, for their contribution to English correction, and literature review.

## Author contributions

**Conceptualization:** Tan-Si Chu, Tan-Huy Chu.

**Data curation:** Tan-Si Chu, Tan-Huy Chu, Tri-Dung Huynh, Hoang-Vu Mai, Van-Dinh Phan.

**Funding acquisition:** Tan-Si Chu.

**Supervision:** Tan-Si Chu, Tan-Huy Chu.

**Formal analysis:** Tan-Huy Chu.

**Investigation:** Tan-Huy Chu, Hoang-Vu Mai, Van-Dinh Phan, Bao-Ngoc Dang, Quoc-Dat Tran, Xuan-Sang Le, Duy-Quang Phan, Anh-Dung Nguyen, Dinh-Hoan Duong.

**Methodology:** Tan-Huy Chu.

**Project administration:** Tan-Huy Chu.

**Resources:** Tan-Huy Chu.

**Software:** Tan-Huy Chu.

**Validation:** Tan-Huy Chu.

**Visualization:** Tan-Huy Chu.

**Writing – original draft:** Tan-Huy Chu.

**Writing – review & editing:** Tan-Huy Chu.

## References

[1] Richter GT Friedman AB. Hemangiomas and vascular malformations: current theory and management. Int J Pediatr. 2012;2012:645678. 22611412 10.1155/2012/645678 PMC3352592

[2] Hartel A Frommherz L Giehl K Häberle B. Vascular malformation and tumors. MMW Fortschr Med. 2023;165:53–5. 10.1007/s15006-023-3097-3 37973754

[3] Carqueja IM Sousa J Mansilha A. Vascular malformations: classification, diagnosis and treatment. Int Angiol. 2018;37:127–42. 29424187 10.23736/S0392-9590.18.03961-5

[4] Jafarian M Alizadeh Tabrizi MA Mashhadi Abbas F Torabi ZS. Intramuscular cavernous malformation in the temporalis muscle: diagnosis and treatment of a rare tumor in a rare site. Clin Case Rep. 2023;11:e8267. 38033698 10.1002/ccr3.8267 PMC10682246

[5] Matsui Y Okada S Nakagami Y Fukagai T Matsuda K Aoki T. Primary retroperitoneal cavernous hemangioma: a case report and review of the literature. Urol Case Rep. 2024;54:102691. 38516175 10.1016/j.eucr.2024.102691 PMC10951441

[6] Hou XF Zhao Z-X Liu L-X Zhang H. Retroperitoneal cavernous hemangioma misdiagnosed as lymphatic cyst: a case report and review of the literature. World J Clin Cases. 2023;11:3560–70. 37383918 10.12998/wjcc.v11.i15.3560 PMC10294187

[7] Debaibi M Sghair A Sahnoun M. Primary retroperitoneal cavernous hemangioma: an exceptional disease in adulthood. Clin Case Rep. 2022;10:e05850. 35592049 10.1002/ccr3.5850 PMC9097370

[8] Cohen MM Jr. Vascular update: morphogenesis, tumors, malformations, and molecular dimensions. Am J Med Genet A. 2006;140:2013–38. 16958055 10.1002/ajmg.a.31333

[9] Kunimoto K Yamamoto Y Jinnin M. ISSVA classification of vascular anomalies and molecular biology. Int J Mol Sci . 2022;23:2358. 35216474 10.3390/ijms23042358 PMC8876303

[10] Chu TS Chu T-H Huynh T-D Phan V-D Dang BN Tran QD. Radical resection of trigeminal schwannoma at the cerebellopontine angle with support of the digital robotic exoscope Synaptive Modus V system: a case report and literature review. Medicine (Baltim). 2023;102:e33492. 10.1097/MD.0000000000033492 PMC10082241 37026917

[11] Chu TS Chu T-H Huynh T-D. Laparoscopic cholecystectomy induce tension pneumocephalus in a patient with ventriculoperitoneal shunt: a case report and literature review. Medicine (Baltim). 2023;102:e35967. 10.1097/MD.0000000000035967 PMC10637415 37960800

